# Myo1f has an essential role in γδT intraepithelial lymphocyte adhesion and migration

**DOI:** 10.3389/fimmu.2023.1041079

**Published:** 2023-05-03

**Authors:** Irving Ulises Martínez-Vargas, Maria Elena Sánchez-Bello, Carlos Emilio Miguel-Rodríguez, Felipe Hernández-Cázares, Leopoldo Santos-Argumedo, Patricia Talamás-Rohana

**Affiliations:** ^1^ Departamento de Infectómica y Patogénesis Molecular, Centro de Investigación y de Estudios Avanzados del Instituto Politécnico Nacional, Mexico City, Mexico; ^2^ Departamento de Biomedicina Molecular, Centro de Investigación y de Estudios Avanzados del Instituto Politécnico Nacional, Mexico City, Mexico; ^3^ Departamento de Biología Celular, Centro de Investigación y de Estudios Avanzados del Instituto Politécnico Nacional, Mexico City, Mexico

**Keywords:** intraepithelial lymphocytes, class I myosins, integrins, migration, cytoskeleton, signaling

## Abstract

γδT intraepithelial lymphocyte represents up to 60% of the small intestine intraepithelial compartment. They are highly migrating cells and constantly interact with the epithelial cell layer and lamina propria cells. This migratory phenotype is related to the homeostasis of the small intestine, the control of bacterial and parasitic infections, and the epithelial shedding induced by LPS. Here, we demonstrate that Myo1f participates in the adhesion and migration of intraepithelial lymphocytes. Using long-tailed class I myosins KO mice, we identified the requirement of Myo1f for their migration to the small intestine intraepithelial compartment. The absence of Myo1f affects intraepithelial lymphocytes’ homing due to reduced CCR9 and α4β7 surface expression. *In vitro*, we confirm that adhesion to integrin ligands and CCL25-dependent and independent migration of intraepithelial lymphocytes are Myo1f-dependent. Mechanistically, Myo1f deficiency prevents correct chemokine receptor and integrin polarization, leading to reduced tyrosine phosphorylation which could impact in signal transduction. Overall, we demonstrate that Myo1f has an essential role in the adhesion and migration in γδT intraepithelial lymphocytes.

## Introduction

Intraepithelial lymphocytes are cells that reside in the epithelial cell layer of the mucosa. Intestinal intraepithelial lymphocytes are mainly T lymphocytes, either αβT or γδT. These cells are sub-classified into conventional T lymphocytes, CD4 and CD8αβ αβT, and unconventional CD8αα αβT and γδT (1). The most unconventional T cells are γδT lymphocytes which constitute up to 60% of the intraepithelial lymphocytes in the small intestine (2–4). These lymphocytes were described as anchored cells in the intestinal epithelium (5). However, seminal works have shown that intestinal intraepithelial γδT lymphocytes migrate between the intestinal epithelium and the basal lamina in the small intestine (6–10). This migration was related to the homeostasis of the intestinal epithelium (9), the control of infections by *Salmonella* and *Toxoplasma gondii* (7, 8), and the pathological shedding (11).

Intraepithelial lymphocyte homing depends mainly on CCR9 and α4β7 integrin (12–14). CCL25 is a cytokine that is produced mainly by epithelial cells in the small intestine, and is the only CCR9 ligand described, and responsible for IEL recruitment (12, 15). In addition, the interstitial migration of these cells depends on other molecules such as occludin (6), integrin αE (16), orphan receptors such as GRP18 (17) and GPR55 (9), and IL-15 (18).

Class I myosins are monomeric myosins that link the actin cytoskeleton to the plasma membrane through their motor and pleckstrin homology (PH) domains, respectively (19). These myosins are expressed in lymphoid and myeloid cells and have been related to cell adhesion, migration, and vesicular trafficking (20, 21). Among the class I myosins, Myo1e and Myo1f are two long-tailed class I myosins with two additional domains in their tail region. Both contain a proline-rich sequence (TH2) and an SH3 domain that recognizes proline-rich sequences, which allow protein-protein interactions (19, 22).

Myo1e participates in neutrophils and B cells integrin mediated-adhesion and migration (23, 24) as well as spreading (25). Similarly, Myo1f is relevant for neutrophil migration (26–28). Particularly, Myo1f has shown to be relevant for the β2 integrin expression in neutrophils (26), β1 and β7 integrins in mast cells (29), and αVβ3 integrin in macrophages (30); presumably due to alterations in vesicular traffic (26). In this regard, Myo1f deficiency results in defects in IgE- and MRGPRX2-dependent mast cell degranulation and TNF secretion (31). Mechanically, Myo1f is required for Cdc42 activation, and its deficiency leads to decreased cortical actin polymerization (31). Additionally, Myo1f has emerged as an α-tubulin interacting protein relevant in the dynein-mediated Syk and CARD9 protein transport from the membrane to the cytoplasm during antifungal activation in macrophages (32). Finally, Myo1f regulates filopodia dynamics, increasing adhesion and migration (33). Together, previous reports suggest a role of Myo1f in the plasma membrane dynamics, cell adhesion, migration, and vesicular trafficking.

Here, we show that intraepithelial lymphocytes express Myo1e and Myo1f. Furthermore, the absence of Myo1e and Myo1f impacts the homing of intraepithelial γδT lymphocytes, but Myo1f shows a more significant impact. This deficiency was due to a reduction in the surface expression of intestinal homing receptors CCR9 and α4β7 integrin, having consequences on the 2D migration of γδT lymphocytes *in vitro*. Mechanistically, Myo1f was relevant for actin-mediated membrane protrusion formation during adhesion and migration. Its absence impacts CCR9 and integrin polarization and leads to tyrosine phosphorylation defects, which could impact in signal transduction.

## Results

### Intraepithelial lymphocytes express long-tailed class I myosins

**Figure 1:**
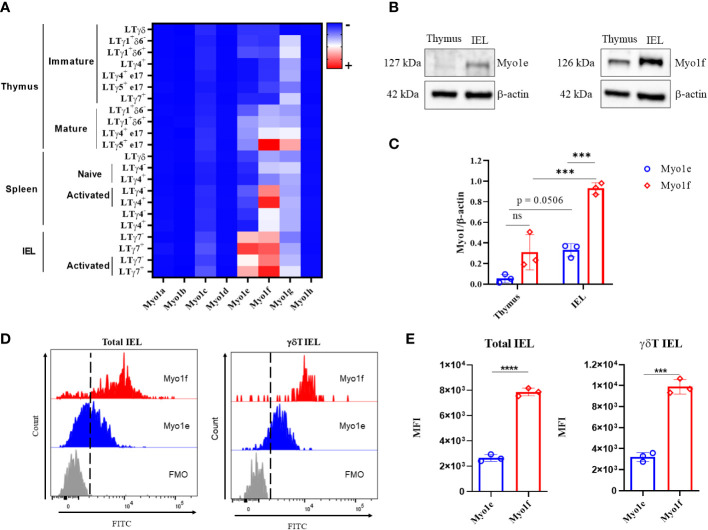
Expression of class I myosins in IEL. **(A)** Heat map of class I myosins expression in γδT lymphocytes from data available at https://www.immgen.org/. **(B)** Cropped western blot of Myo1e (127 kDa) and Myo1f (126 kDa) expression in thymus and total IEL by independent western blots. **(C)** Densitometric analysis of Myo1e and Myo1f expression in thymus and total IEL; β-actin was used as a loading control. A two-way ANOVA test was applied. **(D)** Representative histogram of Myo1e and Myo1f expression in total and γδT IEL by flow cytometry. Fluorescence Minus One (FMO) control. **(E)** Mean Fluorescence Intensity (MFI) of Myo1e and Myo1f expression by flow cytometry. An unpaired t-Student test was applied. Heat map generated with Prism version 8. The western blot represents three independent experiments. Flow cytometry data are from three independent experiments. p-value; ***=0.001, ****=0.0001.

There is no evidence of class I myosin protein expression by intraepithelial lymphocytes (IEL). As a first approach, mRNA class I myosins expression was analyzed in intraepithelial, thymus, and spleen γδT lymphocytes with data from The Immunological Genome Project (https://www.immgen.org/). Heat map reveals that among class I myosins, Myo1e and Myo1f are expressed mainly by intraepithelial γδT lymphocytes, both Vγ7+ and Vγ7-. Myo1f was also expressed in activated spleen Vγ4+ and Vγ4- γδT lymphocytes but did not in resting spleen γδT lymphocytes. Mature thymic Vγ5 γδT lymphocytes also express Myo1f. Whereas the rest of class I myosins remain less represented in γδT lymphocytes (**Figure 1A**). These bioinformatic data suggest that Myo1f is the main class I myosin expressed in γδT lymphocytes. Next, the expression at the protein level of Myo1e and Myo1f was confirmed by western blot. Myo1e and Myo1f are less expressed in thymus cells than in total IEL (**Figure 1B**). Densitometric analysis based on the housekeeping protein β-actin, suggests that both Myo1e and Myo1f relative expression is more abundant in IEL than in thymus. (**Figure 1C**). When the levels of Myo1e and Myo1f in IEL were comparatively analyzed, densitometric analysis of myosins and actin showed a higher ratio for Myo1f compared to Myo1e (**Figure 1C**). These results could suggest that there is a relative higher expression of Myo1f in comparison with Myo1e. Flow cytometry analysis of Myo1e and Myo1f expression showed similar results. Analysis of total IEL showed similar results in γδT and αβT IEL (**Figure 1D** and **Supplementary Figures 1A, B**), whereas thymic and spleen γδT lymphocytes do not express Myo1e but do express Myo1f; however, in less proportion than IEL (**Supplementary Figures 1C, D**). Mean Fluorescence Intensity (MFI) analysis also suggests that Myo1f has a higher relative expression than Myo1e in total IEL and γδT IEL (**Figure 1E**). Thus, intraepithelial lymphocytes express long-tailed class I myosins.

### Myo1f deficiency has an impact on small intestinal intraepithelial lymphocyte counts

**Figure 2:**
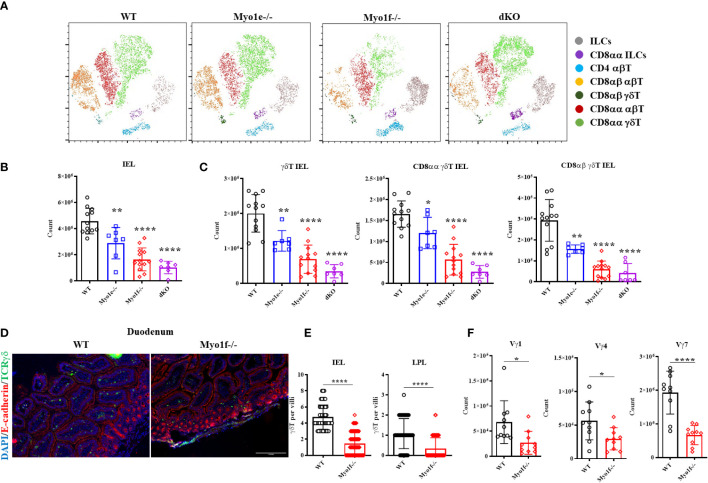
Intraepithelial lymphocyte count in WT, Myo1e-/-, Myo1f-/-, and dKO mice. **(A)** t-SNE algorithm analysis from flow cytometry data in WT, Myo1e-/-, Myo1f-/-, and dKO mice. **(B)** Total and **(C)** γδT, CD8αα γδT, and CD8αβ γδT IEL count in WT, Myo1e-/-, Myo1f-/-, and dKO mice. An unpaired t-Student test was applied. **(D)** Representative histofluorescense staining of γδT lymphocytes in duodenum from WT and Myo1f-/-. **(E)** γδT IEL and Lamina propria lymphocytes (LPL) count in histofluorescense sections. Kolmogorov-Smirnov test was applied. **(F)** Vγ-specific γδT IEL subsets in WT and Myo1f-/- mice. An unpaired t-Student was applied. In flow cytometry analysis, each dot represents one mouse. In tissue count, each dot represents villi and shows pooled data from 3 independent experiments. p-value; *=0.05, **=0.005, ****=0.0001.

IEL were obtained from Myo1e-/-, Myo1f-/-, and dKO mice and were stained for flow cytometry analysis to gain clues about the functions of long-tailed class I myosins. First, the t-SNE (t- Stochastic Neighborhood Embedding) algorithm shows that Myo1e did not considerably affect the proportions of IEL. However, Myo1f deficiency negatively affected CD8αα γδT, CD8αβ, and CD8αα αβT IEL while positively affecting CD4 αβT cells and ILCs. In contrast, the absence of both myosins affected CD8αβ αβT negatively, and CD8αα ILCs positively. Unexpectedly, CD8αα γδT IEL proportion was positively affected in the absence of both myosins (**Figure 2A**). To confirm previous data, conventional gate strategy analysis, and IEL counts were performed (**Supplementary Figure 2A**). Total IEL were reduced in Myo1e-/-, Myo1f-/- and dKO mice (**Figure 2B**). This reduction was also observed for total, CD8αα, and CD8αβ γδT IEL as well as CD8αα, CD8αβ, and CD4 αβT cells (**Figure 2C** and **Supplementary Figure 2B**). Moreover, the reduction was more prominent in Myo1f than Myo1e, but dKO mice do not have an additive effect. In agreement with the bioinformatics and t-SNE analysis, this suggests that Myo1f has a more relevant role in IEL functions. Fluorescence staining of the small intestine tissue sections confirmed a reduction in TCRγδ+ lymphocytes in the epithelial cell layer and lamina propria in Myo1f-/- mice (**Figures 2D, E**). Finally, variable-specific flow cytometry analysis of γδT IEL showed that Myo1f deficiency affects Vγ1, Vγ4, and Vγ7 γδT IEL; however, the reduction was more prominent in the Vγ7 subset in agreement with IEL repertory and Myo1f expression (**Figures 1A**, **2F** and **Supplementary Figure 1C**). Thereby, Myo1f deficiency affects IEL numbers in the small intestine.

### Myo1f absence affects gut homing receptors and integrin expression

**Figure 3:**
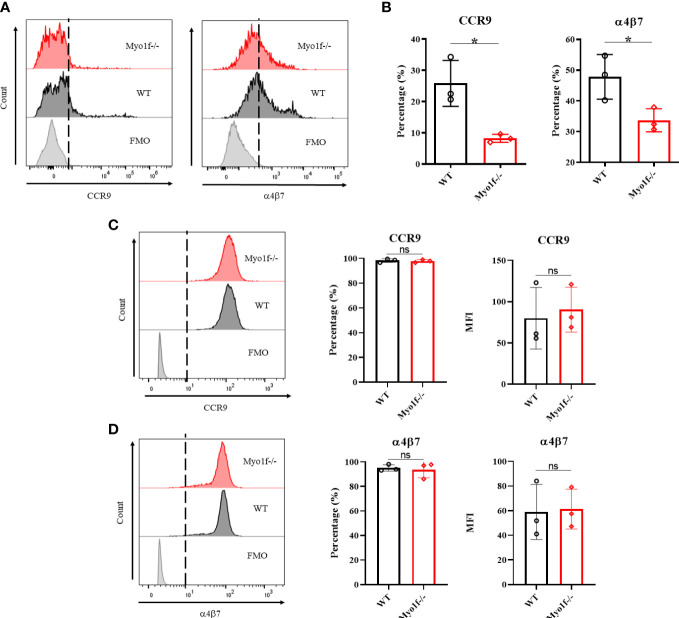
Gut homing and integrin expression. **(A)** Representative histogram of surface CCR9 and α4β7 integrin expression in WT vs. Myo1f-/- γδT cells. **(B)** Percentage of CCR9 and α4β7 positive cells. An unpaired t-Student test was applied. **(C)** Representative histograms of total CCR9 staining and percentage and MFI of CCR9 staining. **(D)** Representative histograms of total α4β7 integrin staining and percentage and MFI of α4β7 integrin staining. For CCR9 and α4β7 MFI analysis, unpaired t-Student tests were applied. Each dot represents one mouse of three independent experiments. p value *=0.05. ns, no significance.

The homing and further localization of IEL in the small intestine is CCR9 and α4β7 integrin-dependent (13, 14). Therefore, CCR9 and α4β7 integrin expression were evaluated in thymic and γδT IEL from WT and Myo1f-/- mice to identify possible defects in gut homing receptors. Although thymic γδT lymphocytes do not have CCR9 and α4β7 surface expression changes, γδT IEL positive to CCR9 and α4β7 were reduced in Myo1f-/- (**Figures 3A, B** and **Supplementary Figures 3A, B**). This reduction suggests not *de novo* synthesis defect because total CCR9 and α4β7 integrin staining showed similar proportions and MFI in WT and Myo1f-/- γδT IEL (**Figures 3C, D**). Furthermore, because Myo1f has been associated with integrin expression, we also evaluated the αE, αLβ2, β1, and αM expression (**Supplementary Figure 3D**). A reduction in αLβ2+, αL+, and β2+ γδT IEL in Myo1f-/-was found. In contrast, only the membrane level of αL was affected (**Supplementary Figures 3E, F**). Hence, Myo1f is required for right gut-homing receptor and integrins expression.

### Myo1f modulates adhesion, spreading, and filopodia formation

**Figure 4:**
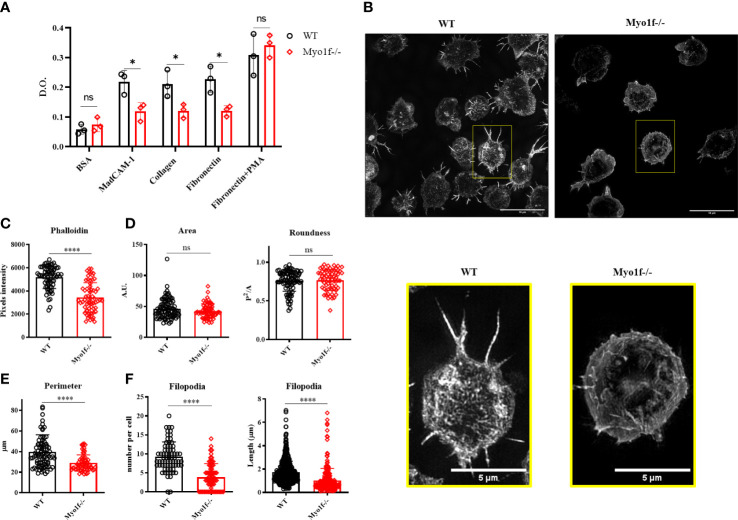
Cell adhesion and spreading. **(A)** Cell adhesion to MadCAM-1, fibronectin, and collagen I. As negative and positive controls, we used 5% BSA and PMA-activated γδT cells, respectively. A two-way ANOVA test was applied. Each dot represents an average of 2 duplicates in 3 independent experiments. **(B)** Representative images of collagen-adhered (spreading) γδT cells for 30 min, stained post-fixation with phalloidin (dilution 1:500) and analyzed by structured illumination microscopy (SIM). **(C)** Phalloidin pixels intensity. **(D)** Cell morphologic analysis of the area, roundness, and **(E)** Perimeter. **(F)** Filopodia count per cell and length. Kolmogorov-Smirnov test was applied. Each dot represents one cell. 90 cells were analyzed. Pooled data from 3 independent experiments. p-value; *=0.05, ****=0.0001. ns, no significance.

Integrins are relevant in cell adhesion. Cell adhesion to recombinant MadCAM-1Fc, fibronectin, and collagen was analyzed to evaluate the consequence of the integrin surface expression reduction. In agreement with reduced integrin surface expression, cell adhesion to MadCAM-1, fibronectin, and collagen was reduced in Myo1f deficient γδT cells, whereas PMA activated-γδT cells adhesion, from the same mice, showed no differences (**Figure 4A** and **Supplementary Figure 4**), thus, indicating defects in integrin-mediated adhesion. Cytoskeleton polymerization was analyzed in collagen-adhered cells (spreading) by structured illumination microscopy (SIM) to gain clues about Myo1f role in integrin-mediated adhesion. Myo1f-/- γδT cells showed less actin polymerization, and fewer membrane projections (**Figure 4B**). MFI of phalloidin staining confirmed less actin polymerization in Myo1f-/- (**Figure 4C**). Morphologic analysis showed no alterations in the area and roundness of the cells. However, a reduced cellular perimeter was observed (**Figures 4D, E**). Reduced perimeter results from shorter filopodia length formation in Myo1f deficient γδT cells (**Figure 4F**). These results suggest that Myo1f modulated cell adhesion, spreading, and filopodia formation.

### Myo1f has a role in migration and lamellipodia formation

**Figure 5:**
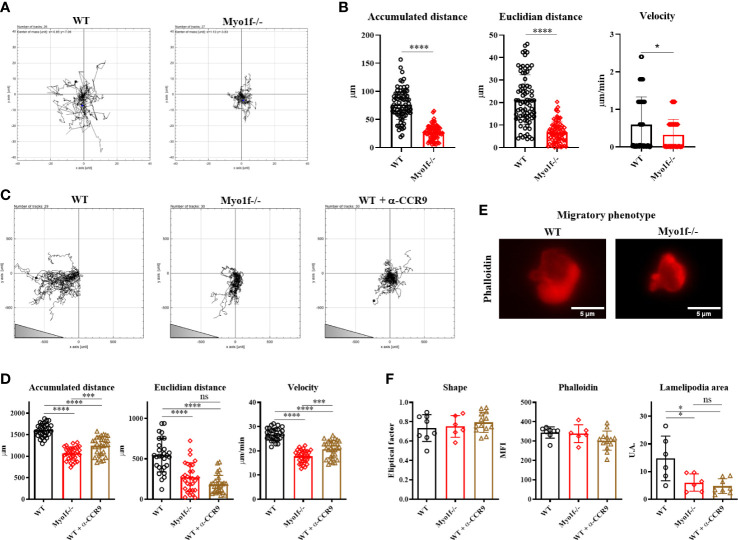
Random and CCL25-dependent migration. **(A)** Representative single-cell migration tracks in random migration. **(B)** Accumulated and Euclidian distance and velocity. **(C)** Representative single-cell migration tracks in CCL25-dependent migration. Triangle represents gradient orientation **(D)** Accumulated and Euclidian distance and velocity in CCL25-dependent migration. **(E)** Phalloidin staining of γδT lymphocyte migratory phenotype; cells were fixed and stained after CCL25-dependent migration. An unpaired t-Student test was applied in distance data, and in velocity data, Kolmogorov-Smirnov test was applied. **(F)** Phalloidin MFI fixed and stained post-migration, shape factor, and lamellipodia area of γδT lymphocyte migratory phenotype. For random and CCL25-dependent migration, 80 and 50 cells were analyzed, respectively. For migratory phenotype, 10 cells were analyzed. A one-way ANOVA test was applied. p-value; *=0.05, ***=0.0005, ****=0.0001. ns, no significance.

To determine if Myo1f has a role in γδT cell migration, we evaluated 2D random and CCL25-dependent migration. In the absence of CCL25, Myo1f -/- cells showed reduced random migration (**Figure 5A** and **Supplementary movies 1, 2**), denoted by a reduced accumulated (total distance traveled between two points) and Euclidian (straight line distance between two points) distance, and velocity (**Figure 5B**). CCL25-dependent migration showed a gradient-specific migration by WT cells. The CCL25-dependent migration by WT cells was reduced with an anti-CCR9-blocking antibody. Myo1f-deficient cells showed reduced migration to the CCL25 gradient, consistently with a reduced expression of CCR9, and comparable to CCR9-blocked WT cells (**Figure 5C** and **Supplementary movies 3-5**).

Myo1f-/- γδT cells demonstrated reduced accumulated and Euclidian distance and velocity (**Figure 5D**). Myo1f-/- γδT cells showed a similar shape and total actin polymerization. However, they had a reduced lamellipodia area compared with WT γδT cells (**Figures 5E, F**). Thus, Myo1f is required in random and chemokine-dependent migration and lamellipodia formation.

### Myo1f regulates CCR9 and α4β7 polarization and signaling

**Figure 6:**
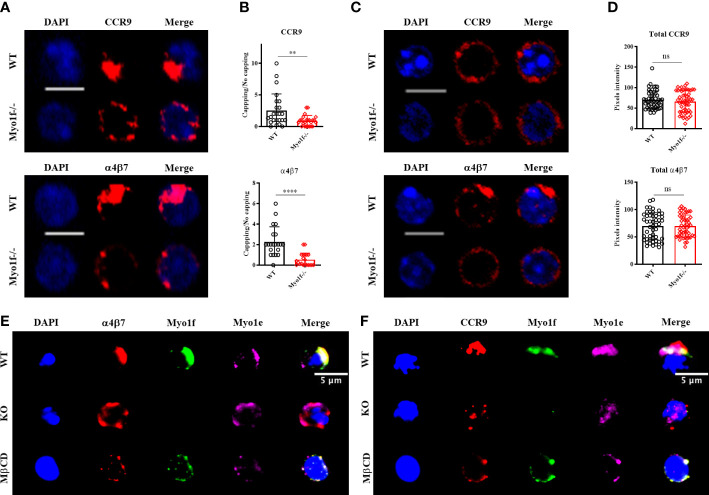
Myo1f role in CCR9 and α4β7 integrin polarization. **(A)** Representative confocal images of CCR9 and α4β7 capping induction in WT and Myo1f-/- γδT IEL. **(B)** Capping/no capping quotient of CCR9 and α4β7 defined by the molecule agglomeration (32). Each dot represents capping/no capping quotient per field (3-6 cells per field, 25 fields per group). Mann-Whitney test was applied. Pooled data from 3 independent experiments. **(C)** Representative confocal images of total CCR9 and α4β7 staining in WT and Myo1f-/- γδT IEL **(D)** Pixels intensity of total CCR9 and α4β7 staining. The Kolmogorov-Smirnov test was applied. Pooled data from 3 independent experiments. **(E)** Representative epifluorescence images of α4β7 capping phenotypes from WT, Myo1f-/- and MβCD pre-treated γδT IEL. **(F)** Representative epifluorescence images of CCR9 capping phenotypes from WT, Myo1f-/-, and MβCD pre-treated γδT IEL. Scale bar 5 μm. p-value; **= 0.005, ****=0.0001. ns, no significance.

To elucidate the defects in the mechanism of adhesion and migration in Myo1f-/- γδT IEL, we evaluated the CCR9 and α4β7 integrin polarization by capping assays (34). WT γδT IEL polarizes CCR9 receptor and α4β7 integrin in large patches, whereas Myo1f-/- γδT IEL showed smaller scattered patches (**Figure 6A**). To define the capping, we employed an index between cap length and cell circumference, as previously reported (34).

The ratio of cells with capping/uncapping images confirms that Myo1f deficient cells fail to polarize CCR9 and α4β7 (**Figure 6B**). Surface expression of CCR9 and α4β7 was reduced in Myo1f defective cells compared to WT concordantly with flow cytometry data (**Supplementary Figures 5A, B** and **Figures 3A, B**). In contrast, total staining remains identical, suggesting no defects in *de novo* synthesis of both molecules (**Figures 6C, D**).

Previously, Myo1g has shown accumulation in clustering sites of adhesion molecules (34). To investigate where long-tailed class I myosin localize during the capping process Myo1e and Myo1f staining was performed. Myo1e and Myo1f localize in CCR9 and α4β7 in WT γδT IEL, whereas Myo1e remains adjacent to the capping site in α4β7 but has a diffuse localization in the CCR9 capping site in Myo1f-/- γδT IEL (**Figures 6E, F**). This result suggests that both Myo1e and Myo1f could participate in receptor polarization at the plasma membrane. Protein clustering in the cell membrane requires cholesterol and sphingolipid-enriched liquid-ordered microdomains called lipid rafts (35). To evaluate the role of lipids rafts in the capping process and in Myo1e and Myo1f localization, WT γδT IEL were preincubated with methyl-β-cyclodextrin (MβCD) before capping assay. MβCD phenotype resembles Myo1f-/- smaller and scattered patches of CCR9 and α4β7 (**Figures 6E, F**). In contrast, both myosins remain localized at the capping site at CCR9 and α4β7 (**Figures 6E, F**). This result suggests that long-tailed class I localization at the capping site does not depend on the lipid raft ensemble, whereas the capping of CCR9 and α4β7 is lipid raft dependent.

CCR9 and α4β7 clustering, as well as lipid raft, are essential in signal transduction (35).

**Figure 7:**
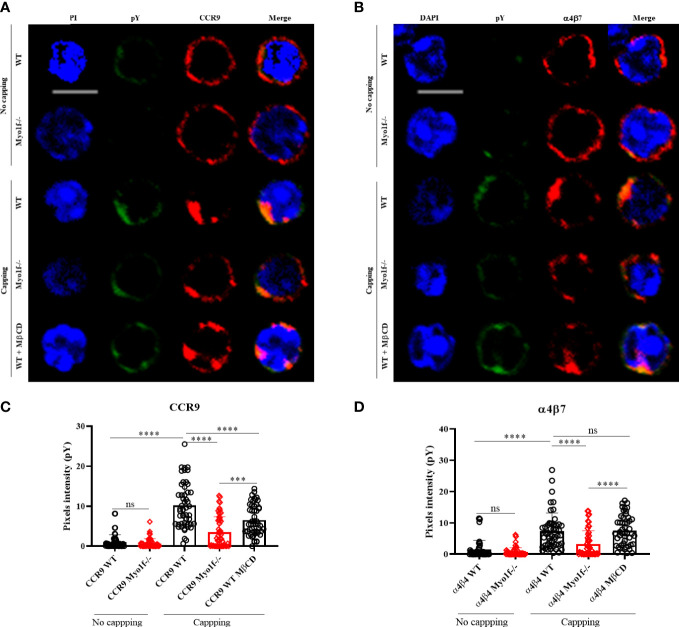
Myo1f deficiency in tyrosine phosphorylation-mediated signaling. **(A)** Representative CCR9 no capping and capping phenotype and phosphotyrosine staining from WT, Myo1f-/- and MβCD pre-treated γδT IEL **(B)** Representative α4β7 no capping and capping phenotype and phosphotyrosine staining from WT, Myo1f-/- and MβCD pre-treated γδT IEL **C)** Phosphotyrosine pixels intensity in WT, Myo1f-/- and MβCD pre-treated γδT IEL in CCR9 capping phenotype. A Kruskal-Wallis test was applied. Each dot represents one cell. Pooled from 3 independent experiments. **(D)** Phosphotyrosine pixels intensity in WT, Myo1f-/- and MβCD pre-treated γδT IEL in α4β7 capping phenotypes. The Kruskal-Wallis test was applied. Each dot represents one cell. Pooled data from 3 independent experiments. Scale bar 5 μm. p-value; ***=0.0005, ****=0.0001. ns, no significance.

As a general approach to analyze whether Myo1f participates in signaling pathways activation, tyrosine phosphorylation induced by CCR9 and α4β7 was evaluated. Incubation with anti-CCR9 and anti-α4β7 antibodies, without capping, induces low tyrosine phosphorylation levels. In contrast, the capping triggered by anti-CCR9 and anti-α4β7 antibodies induce a high level of tyrosine phosphorylation. As expected, deficient-polarization clustering by Myo1f-/- cells resulted in reduced phosphotyrosine detection. Similarly, the treatment of WT cells with MβCD, resembled Myo1f-/- reduced tyrosine phosphorylation (**Figures 7A, B**). As a control, anti-CD3ε antibody-induced phosphorylation showed no differences between WT and Myo1f-/- cells (**Supplementary Figures 5C, D**). Quantitation of pixels intensity confirmed less tyrosine phosphorylation induced by CCR9 and α4β7 capping in Myo1f-/- deficient cells. However, with anti-α4β7 the treatment with MβCD did not resemble the capping phenotype of Myo1f-/- cells, as it did with anti-CCR9 (**Figures 7C, D**), suggesting different lipid raft requirements between CCR9 and α4β7 induced phosphorylation. Thus, Myo1f regulates CCR9 and α4β7 clustering in the plasma membrane to induce tyrosine phosphorylation that could impact signal transduction.

## Discussion

Class I myosins have been related to migration, adhesion, and vesicular trafficking in leukocytes (21, 22). However, their role in T lymphocytes remains poorly studied. This work addresses the role of long-tailed class I myosins in the adhesion and migration of intraepithelial lymphocytes.

Here, we demonstrated that Myo1e and Myo1f are expressed at the protein level by αβT and γδT intraepithelial lymphocytes. Despite the differences of the two antibodies employed to detect Myo1e and two others to detect Myo1f, our results demonstrate that the relative expression of Myo1f was always higher than Myo1e in these cells. Which agrees with mRNA expression from the Immunological Genome Project (https://www.immgen.org/). However, the possibility that the difference between the antibodies employed may contributes to the differences observed between Myo1e and Myo1f expression can not be ruled out. Consistently to the data available in the Immunological Genome Project, neither thymic nor spleen γδT cells express Myo1e, but ~25% of cells express Myo1f. Additionally, activated γδT cells increased Myo1f expression compared to their naïve counterpart. These suggest that long-tailed class I myosins could be expressed after activation or differentiation. It has been previously demonstrated that monocytes acquire Myo1f expression upon macrophage differentiation (30). At the same time, Myo1e deficiency mainly affects activated B cells (24). In this regard, Taspase1 and Myo1f inversely correlate with differentiation in the stem cell-monocyte-macrophage lineage (33). Mechanistically, Myo1f is cleaved by the caspase 1 protease, which impaired its function, indicating a Tasp1 negative regulation of Myo1f during differentiation (33). As effector cells, γδT intraepithelial lymphocytes express more Myo1e and Myo1f than their thymus and spleen counterpart.

The t-SNE algorithm analysis showed modifications in the proportions of IEL subpopulations in Myo1e-/-, Myo1f-/-, and dKO mice. Some populations increased such as CD4 αβT and ILCs in Myo1f-/-, whereas some others reduced their proportions, such as CD8αβ αβT. Unexpectedly, CD8αα γδT cells increased their percentages in dKO mice. Some explanations for the molecular mechanisms that would account for the differences behind the increased or decreased proportion of IEL subpopulations would include, for example; populations less affected by these ratios could indicate less dependence on the expression of Myo1e, Myo1f, or both, causing differences in cellular ratios (36), gut epithelial renewal and repair may directly affect the functions of the intestinal epithelium, and these processes could affect the function or proportions of the different IEL subpopulations (37, 38), the proportion of IEL in the intestinal compartment depends on recruitment and their proliferative capacity (39, 40); therefore, variations in IEL proportions could be due to different proliferation rates, since Myo1e- and Myo1f-deficient mice are complete KO mice and not conditioned or induced, there is the possibility of a compensatory effect during mouse development, in which some IEL populations occupy the vacated niche (41, 42). However, the total counts of IEL were consistently lower in Myo1e-/-, Myo1f-/-, and dKO than in the WT mice. Therefore, the difference in the proportions does not reflect the significant reduction of all intraepithelial lymphocytes in the small intestine in all deficient mice tested in this work. Interestingly, only Myo1f-/- mice have a decreased γδT lymphocyte proportion, whereas Myo1e-/- mice remain identical to WT. This difference could be due to Myo1f showing higher relative expression than Myo1e. Additionally, Myo1e and Myo1f are different in the 950-1000 amino acid region, where the TH2 domain is located, and thus, this could imply different protein-protein interactions (https://www.uniprot.org/). However, this needs to be experimentally confirmed. Despite this, dKO mice have no additive effects in the γδT cell proportion. It has been demonstrated that dKO macrophages showed increased actin waves and Arp2/3 recruitment during Fc-mediated phagocytosis (43), suggesting altered actin polymerization. If the long-tailed class I myosin deficiency is compensated by other class I myosins requires to be addressed. Based on these results, we focused on understanding the Myo1f function in γδT intraepithelial lymphocytes.

The α4β7 integrin is essential in gut-specific recruitment (14). β7-/- mice have neither intraepithelial lymphocytes, αβT, nor γδT in the gut (44). Myo1f-/- γδT intraepithelial lymphocytes express less surface α4β7 integrins. Similarly, the knock-down of Myo1f in mast cells reduces β7 integrin expression (29). Contrary to the first description of Myo1f deficiency in neutrophils (26), αLβ2 integrin was decreased in Myo1f-/- γδT intraepithelial lymphocytes. This difference could be explained due to the cell type or activation status. Moreover, β2-/- mice have no defects in γδT or αβT intraepithelial lymphocytes count (45). We did not observe significant differences in β1 and αM expression. However, β1 low expression was reported in Myo1f knock-down mast cells (29), while αVβ3 integrin expression is enhanced in stably-transfected macrophages with Myo1f-GFP (30). Our results provide evidence of the relevant role of Myo1f in integrin expression.

Due to decreased integrin surface expression, Myo1f-/- γδT IEL showed less adhesion to MadCAM-1 (ligand of α4β7), fibronectin, and collagen. Mechanically, Myo1f-/-deficiency results in fewer filopodia formation. This filopodia formation depends mainly on the actin cytoskeleton polymerization dependent on the activity of Cdc42 (46). In agreement, reduced Cdc42 activation was found in resting and IgE-mediated exocytosis (31).

Intraepithelial lymphocyte homing depends on CCR9, and its deficiency reduces γδT proportion (12, 13). Similarly, CCL25 deficiency showed a similar reduction (12). We demonstrated that Myo1f-/- deficiency results in less CCR9 surface expression. 2D migration assays showed less accumulated and Euclidian distance and velocity in random and CCL25-dependent migration in Myo1f-/- cells. These results suggest that Myo1f is required for random and chemokine-dependent migration. The two-dimensional migration assays were performed using Ibidi μ-Slide Chemotaxis chambers coated with collagen IV, in which problems with cell adhesion and flow, among others, can occur. Therefore, we can not rule out the possibility that the effect observed in migration assays may be underestimated or overestimated. Despite this, our results on the role of Myo1f in cell migration are consistent with what has been previously reported. Myo1f-/- cells have reduced lamellipodia area in the CCL25-dependent migration, possibly due to a reduction in the Rac1 activation. Rac1 regulates lamellipodia formation by actin polymerization during migration (46). Myo1e-/- B cells stimulated with CXCL12 show reduced Rac1 activation (24). However, the precise mechanism by which Myo1f-dependent Rho GTPases activation occurs is unknown. Myo1f interacts with 3BP2 (29), an adapter molecule necessary in neutrophil migration (47) which is essential in Rho GTPase family activation by modulating VAV1 activation (48), suggesting that Rho GTPase activation defects in Myo1f deficiency could be due to VAV reduced activation. This way, long-tailed class I myosins are relevant for the Rho GTPase family activation.

Typically, class I myosin localize in the plasma membrane and colocalizes with cortical actin. This localization has been related to membrane tension regulatory properties. In this regard, it has been reported that Myo1g controls plasma membrane tension in B cells (49). Additionally, Myo1g participates in lipid raft-CD44 mobilization and recycling (34). Here we demonstrate that Myo1f deficiency impaired correct CCR9 and α4β7 polarization. Moreover, Myo1e and Myo1f re-localize at the capping site, indicating their participation in this process. Furthermore, cholesterol depletion with MβCD in WT cells resembles Myo1f-/- capping phenotype suggesting lipid raft-mediated polarization. Despite impaired CCR9 and α4β7 polarization in MβDC preincubation, Myo1f localization remains at the capping site, suggesting that Myo1f localization is lipid raft-independent. However, MβCD disrupts Myo1e localization at the CCR9 capping site. The specific phosphoinositide binding by Class I myosins could explain the differences in the patterns observed between Myo1e, and Myo1f in γδT intraepithelial lymphocytes (50).

Finally, we demonstrated that altered CCR9 and α4β7 polarization reduce tyrosine phosphorylation compared to the WT counterpart. Together, this work provides evidence about the role of Myo1f in adhesion and migration in γδT intraepithelial lymphocytes, mediating the lipid raft dependent-CCR9 and α4β7 polarization that could affect cell signaling.

## Material and methods

### Mice

Female (6-8 weeks old) C57BL/6 wild type, Myo1e-/- (B6.129S6(Cg)-*Myo1e^tm1.1Flv^*/J), Myo1f-/- (B6.129S6-*Myo1f^tm1.1Flv^*/J) and Myo1e-/-+Myo1f-/- (dKO) (generated in the CINVESTAV by crossing Myo1e-/- with Myo1f-/- during ten generations to obtain double homozygous knock out) mice were bred and maintained at CINVESTAV-UPEAL (Unit of Production and Experimentation with Animals of Laboratory) (Mexico City, Mexico) facilities. Additionally, Myo1f-/- mice were maintained in the Johns Hopkins Bloomberg School of Public Health (Baltimore, Maryland, USA) facilities for some experiments. WT mice were purchased from Jackson Laboratories. Myo1e-/- and Myo1f-/- mice were kindly donated by Dr. Richard Flavell from Yale School of Medicine, New Haven, CT. Mice genotypification was carried out employing the primers described previously (26, 51).

All experiments were approved by the Animal Care and Use Committee at Cinvestav and carried out following ARRIVE (Animal Research: Reporting of *in vivo* experiment) guide.

### Bioinformatic analysis

Class I myosin mRNA expression was obtained from The Immunological Genome Project in the portal https://www.immgen.org. Myo1a (Probe set ID: 10366994), Myo1b (10354432), Myo1c (10378697), Myo1d (10389022), Myo1e (10586781), Myo1f (10443980), Myo1g (10384154) and Myo1h (10524515) γδT lymphocytes RMA (Robust Multiarray Averaging)-normalized mRNA expression value was manually obtained from microarray data using the Gene Skyline browser and graphed as a heat map using Prism Software (Irvine, CA) version 8 in order to show the relative expression.

### Intraepithelial lymphocyte isolation

Complete small intestines were removed from mice, cut longitudinally, and then in 1 cm fragments. Then, fragments were placed in 1 mM DTE (1,4-Dithioerythritol) (Sigma-Aldrich, St Louis, MO.) in HBSS (Hanks Balance Salt Solution) without Ca^+2^ and Mg^+2^ (Thermo Fisher Scientific, Waltham, MA.) for 30 min, twice. The cell suspension was filtered using a 70 μM cell strainer (Thermo Fisher Scientific, Waltham, MA.), transferred to 50 mL tubes, and centrifuged at 1500 rpm for 10 min. Pellets were washed twice with HBSS and then resuspended in discontinuous 40/70% Percoll^®^ (Sigma-Aldrich, St Louis, MO.) gradient and centrifuged at 2500 rpm for 30 min (52). The interface was collected and washed two times.

### Flow cytometry

IEL were incubated with anti-CD16/32 (clone 93, dilution 1:500) antibody (Biolegend, San Diego, CA.) and Live/Dead Aqua (dilution 1:1000) (Thermo Fisher Scientific, Waltham, MA.) for 30 min on ice. Then, IEL were washed and incubated with anti-CD45 (clone 30-F11, dilution 1:500) (eBioscience, Thermo Fisher Scientific, Waltham, MA.), -CD3ε (clone 145-2C11, dilution 1:300) (eBioscience), -TCRγδ (clone GL3, dilution 1:300) (BD Biosciences, Franklin Lakes, NJ.), -CD8α (clone 53-6.7, dilution 1:300) (BD Biosciences), -CD8β (clone YTS156.7.7, dilution 1:300) (Biolegend) and CD4 (clone GK1.5, dilution 1:200) (BD Biosciences) for total IEL analysis. With anti-CD3ε (eBioscience), -TCRγδ (BD Bioscience), -TCRVγ1.1/1.2 (clone 4B2.9, dilution 1:200) (Biolegend), -TCRVγ2 (clone UC3-10A6, dilution 1:200) (BD Biosciences) for Vγ specific γδT IEL analysis. Anti-CCR9 (clone eBioCW-1.2, dilution 1:200) (eBioscience), -α4β7 (clone DATK-32, dilution 1:200) (Invitrogen, Thermo Fisher Scientific, Waltham, MA.), -αE (clone 2E7, dilution 1:200) (eBioscience), -αLβ2 (clone H155-78, dilution 1:200) (Biolegend), -αM (clone M1/70, dilution 1:200) (Biolegend), -β1 (clone HMb1-1, 1:200) (Biolegend), were also employed. For intracellular staining, cells were fixed with 1% paraformaldehyde (PFA) for 5 min. Then the cells were permeabilized with saponin 0.1% for 15 min, next incubated with 10% goat serum (Sigma-Aldrich, St. Louis, MO.) at 37°C, and further incubated with anti -Myo1e (Cat. PAD434Mu01, dilution 1:500, Cloud-Clone Corp, Houston, AZ.), -Myo1f (Cat. 13933-AP, dilution 1:500, Proteintech, Rosemont, IL.), -CCR9 (clone 9B1, dilution 1:100, Biolegend) and α4β7 (clone DATK-32, dilution 1:100, Biolegend) antibodies. For indirect staining, secondary anti-rabbit FITC (dilution 1:1000, Jackson ImmunoResearch Laboratories, West Grove, PA.), anti-rat AF647 (dilution 1:1000, Jackson ImmunoResearch Laboratories, West Grove, PA.), or anti-rabbit Pacific Blue (dilution 1:1000 Jackson ImmunoResearch Laboratories) were employed. Finally, cells were resuspended in PBS and analyzed in Cytoflex LX (Beckman Coulter, Brea, CA.), LSR Fortessa (Becton Dickinson, San Jose, CA.), BD Influx (Becton Dickinson, San Jose, CA.) or Attune Next (Thermo Fisher Scientific, Waltham, MA.) cytometers. Data were analyzed using FlowJo Software (BD Biosciences) version 10.6.

### γδT IEL purification and culture

IEL were incubated with anti CD16/32 for 30 min at 4 °C. Then, cells were incubated with anti-TCRαβ biotin-coupled (H57-597) (Biolegend) for 30 min at 4°C. Next, cells were incubated with anti-biotin beads (Miltenyi Biotec, Bergisch Gladbach) for 15 min at 4°C. Finally, cells were washed, and γδT IEL were negatively selected by an LD MACS column (Miltenyi Biotec). γδT IEL were incubated with Live/Dead Scarlet (Thermo Fisher Scientific) and anti-TCRγδ-PE (Biolegend) antibody for 30 min at 4°C. Cell viability and purity were confirmed by flow cytometry. Cell viability was >95%, and purity was 90%. IEL were cultured as described previously (53). Purified γδT IEL were incubated with RPMI 1640 (Thermo Fisher Scientific) medium supplemented with 10% fetal bovine serum (ATCC, Manassas, VA.), 2.5% HEPES (Thermo Fisher Scientific), 1% glutamine (Thermo Fisher Scientific), 1% Pen/Strep (Thermo Fisher Scientific), 1% sodium pyruvate (Thermo Fisher Scientific), 1% non-essential amino acids (Thermo Fisher Scientific) and 0.2% β-mercaptoethanol (Thermo Fisher Scientific) plus murine recombinant IL-2 (10 U/mL), IL-4 (200 U/mL), IL-3 (100 U/mL), and IL-15 (100 U/mL) (PeproTech, Cranbury, NJ.). After two days, cells were re-plated and cultured only with IL-2 (10 U/mL). The medium was replaced every 3-5 days.

### Western blot

Proteins from total extracts (30 μg) were resolved in 10% SDS-PAGE gels. Proteins were transferred to a nitrocellulose membrane (Bio-Rad, Hercules CA.), and protein transfer was confirmed with Ponceau red. First, membranes were blocked with 5% milk in 0.05% TBS-Tween 20 buffer for 1 h at room temperature. Next, anti-Myo1e (dilution 1:1000, Proteintech) and -Myo1f (dilution 1:1000, Santa Cruz Biotechnology) antibodies were incubated in 2% milk in TBS-T overnight at 4°C on independent membranes; next, membranes were washed and incubated with anti-rabbit HRP antibody (dilution 1:10000, Jackson ImmunoResearch Laboratories) for 1 h at room temperature. The same membranes were then incubated with anti-β actin (dilution 1:2000, Santa Cruz Biotechnology), in 2% milk in TBS-T overnight at 4°C; next, membranes were washed and incubated with anti-mouse HRP antibody (dilution 1:10,000, Jackson ImmunoResearch Laboratories) for 1 h at room temperature. Finally, membranes were washed and revealed with a super signal western blot system (Pierce™, Thermo Fisher Scientific, Waltham, MA.) in C-digit equipment (LI-COR Bioscience, Cambridge, UK.). Densitometric analysis was performed using Image J software (National Institutes of Health, Bethesda, MD.) version 1.52.

### Tissue immunofluorescence staining

Duodenum sections were obtained from WT and Myo1f-/- mice. First, tissue fractions were pulled in optimal cutting temperature media (ProSciTech, Townsville, Queensland, Australia) and frozen in liquid nitrogen. Next, tissue sections (5 μm thick) were placed in poly-L-lysine coated slides, fixed with absolute acetone for 20 min at -20°C, and then blocked with 5% BSA for 1 h at room temperature. Next, tissues were incubated with purified anti-TCRγδ (dilution 1:500, Biolegend) and anti-E-cadherin (dilution 1:100, Santa Cruz Biotechnology, Dallas, TX.) antibodies for 1 h a room temperature. Afterward, tissues were stained with anti-hamster-AF555 (dilution 1:1000, Thermo Fisher Scientific) and anti-rat-FITC (dilution 1:1000, Jackson ImmunoResearch Laboratories) secondary antibodies, respectively. Finally, tissues were incubated with DAPI (dilution 1:10000, Thermo Fisher Scientific) for 10 min and mounted with 10% glycerol. Tissues were analyzed immediately in an Olympus X microscope (Olympus Scientific, Tokyo, Japan). γδT cell count was performed with Image J software (NIH), version 1.52.

### Cell adhesion assay

Plates (96-wells) were pre-coated with murine recombinant MadCAM-1 (R&D Systems, Minneapolis, MN.), fibronectin, collagen, or 5% BSA overnight at 4°C. Then, plates were blocked with 5% BSA in PBS for 1 h a 37°C. Next, 2 x 10^5^ γδT IELs or PMA activated-γδT IEL were seeded and incubated at 37°C for 1 h. After that, non-adhered cells were removed, and plates were washed once with PBS. Then cells were fixed with PFA 4% for 10 min at room temperature. Next, plates were washed and stained with crystal violet for 30 min; plates were washed 5 times and then incubated with 10% SDS for 30 minutes. Finally, plates were read in a 680-microplate reader (Bio-Rad, Hercules CA.) equipment.

### Spreading

24-hours cultured γδT IEL (1 x 10^5^) were seeded in 1.5 mm coverslips previously coated with 5 μg/mL of Collagen I (Corning^®^, Bedford, MA.). Cells were incubated for 1 h at 37°C with 5% CO_2_. Next, cells were fixed with 4% formaldehyde for 30 minutes at room temperature and then permeabilized with 0.2% Triton X-100 (Sigma-Aldrich) for 15 minutes. After that, cells were stained with phalloidin-CF555^®^ (dilution 1:500, Biotium, San Francisco, CA.) for 1 h at 37°C and mounted with ProlongGold with DAPI (Thermo Fisher Scientific). Cells were observed in a GE DeltaVision OMX-SR microscope (GE Healthcare, Chicago, IL). Images were analyzed using Fiji Software (National Institutes of Health, Bethesda, MD.) version 2.3.0.

### 2-dimension migration assay

γδT IEL (2 x 10^5^) were seeded in an μ-Slide Chemotaxis^®^ chamber coated with collagen IV (Ibidi, Martinsried, Munich, Germany) for 30 min at 37°C. Then, non-adhered cells were removed, and the chamber was filled with a complete RPMI-1640 medium. Murine recombinant CCL25 (R&D Systems, Minneapolis, MN.) (100 ng/ml) was added in one slide plug. Slides were immediately placed on the stage of a DeltaVision Elite microscope (GE Healthcare, Chicago, IL). Time-lapse videos were taken every 30 sec for 30 min. Distance and velocity analysis was made with Fiji software (Fiji Software (NIH), version 2.3.0, with the TrackMate plugin (54) and the Chemotaxis and Migration Tool (Ibidi).

### Capping assay

γδT IEL were incubated with purified anti-CCR9 (clone 9B1, dilution 1:100) (Biolegend) or anti α4β7(clone DATK-32, dilution 1:100) antibody for 20 min at 4°C. Cells were washed with PBS and incubated with the anti-rat-AF594 (dilution 1:500, Thermo Fisher Scientific) antibody for 30 min at 37°C to induce cross-linking. A parallel cold incubation was employed as negative control of capping. Cells were washed and then fixed with 4% PFA. For intracellular staining, cells were permeabilized with 0.1% Triton X-100 for 15 min, then incubated with 5% BSA for 1 h, and then incubated with anti- Myo1e (dilution 1:200, Proteintech), anti- Myo1f (clone B-5, dilution 1:200) (Santa Cruz Biotechnology) and anti-phospho-tyrosine (clone PY20, dilution 1:1000) (Biolegend) antibodies. Then, cells were incubated with anti-mouse FITC (dilution 1:1000, Thermo Fisher Scientific) and anti-rabbit AF647 (dilution 1:1000, Biolegend). After staining, cells were mounted in poly-L-lysine (Sigma-Aldrich) pre-coated coverslips with ProlongGold with DAPI. In some experiments, cells were pre-incubated with 5 mM MβCD (Sigma-Aldrich) for 15 min at 37°C. CD3ε (clone 145-2C11) stimulation was employed as positive phosphotyrosine control. For the phosphotyrosine detection, cells were treated with 1 mM vanadate (New England BioLabs, Ipswich, MA.). To define capping, we employed an index between the cap length and the cell circumference as previously reported (34). Cells were observed by confocal microscopy or by epifluorescence microscopy and analyzed with the Fiji Software (NIH), version 2.3.0.

### Statistical analysis

Results shown are mean with +/- SD. The normality of the data was analyzed by Anderson-Darling, D’Agoston & Pearson, Shapiro-Wilk or Kolmogorov test. The specific statistical test, p values and the number of samples or cells (n) used are mentioned in each figure legend.

## Data availability statement

The original contributions presented in the study are included in the article/**Supplementary Material**. Further inquiries can be directed to the corresponding authors.

## Author contributions

Conceptualization: IM-V, LS-A, PTR; Methodology: IM-V, MS-B, CM-R, FH-C; Validation: IM-V, LS-A, PTR; Investigation: IM-V, MS-B, CM-R, FH-C; Resources: LS-A, PTR; Writing (original draft): IM-V; Review and Editing: IM-V, LS-A, PTR; Visualization: IM-V, LS-A, PTR; Supervision: LS-A, PTR; Funding: LS-A, PTR. All authors contributed to the article and approved the submitted version.
