# Supplementary material for: Myo1f has an essential role in γδT intraepithelial lymphocyte adhesion and migration
Source: Front Immunol. 2023 May 3;14:1041079. doi: 10.3389/fimmu.2023.1041079 (PMC10189005; doi:10.3389/fimmu.2023.1041079)
Supplement: Supplementary file 1 [file DataSheet_1.docx]

***Supplementary material***


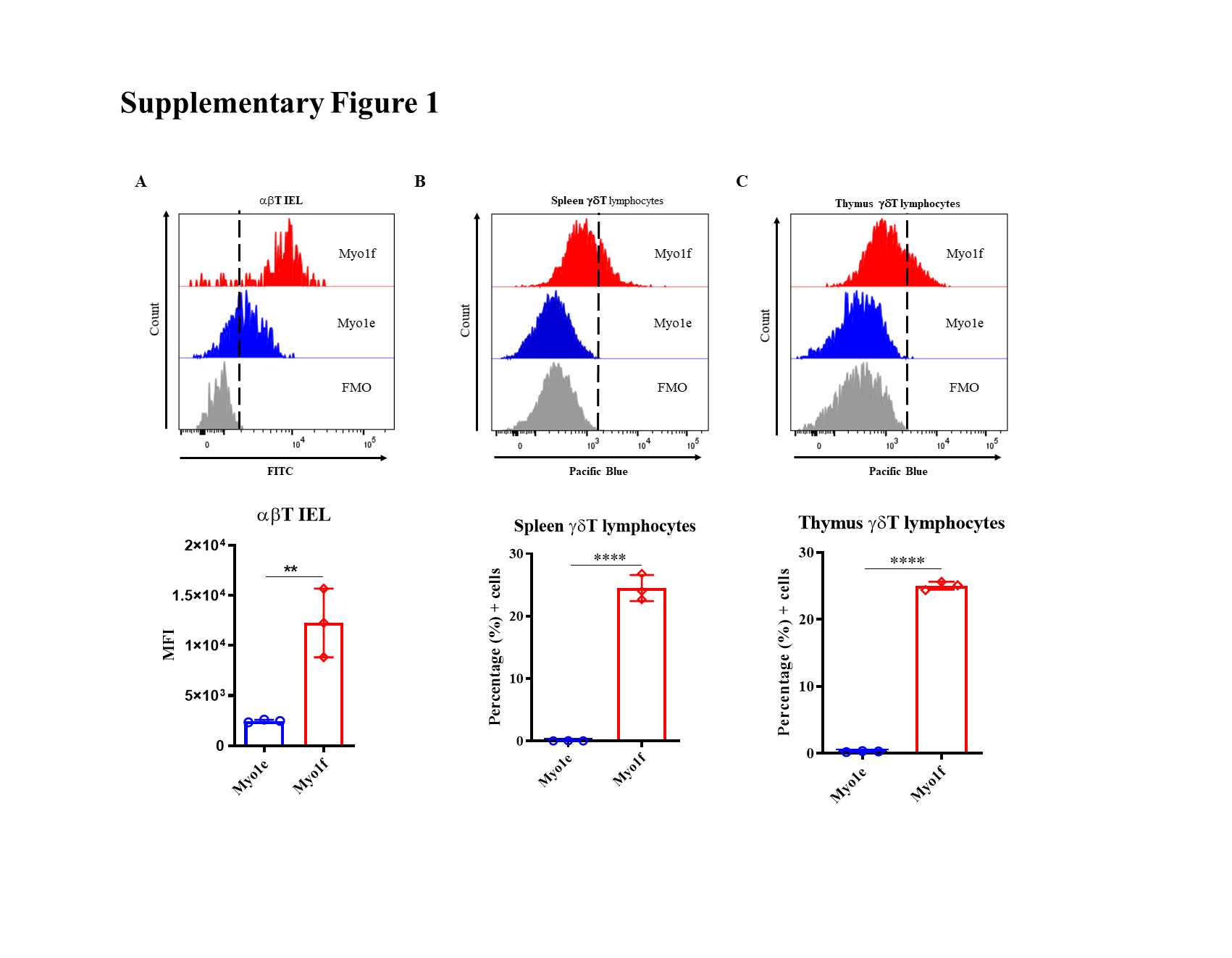


**Supplementary figure 1. Long-tailed class I myosins expression in αβT IEL and thymus and spleen γδT lymphocytes. A)** Representative histogram of flow cytometry analysis of Myo1e and Myo1f expression in αβT IEL. **B)** MFI of Myo1e and Myo1f expression in αβT IEL. **C)** Representative histogram of flow cytometry analysis of Myo1e and Myo1f expression in spleen and thymus γδT lymphocytes. **D)** Percentage of Myo1e and Myo1f positive γδT lymphocytes in the spleen and thymus. Three independent experiments were performed, and *t*-student was applied. p-value; **= 0.0078, ****=0.0001


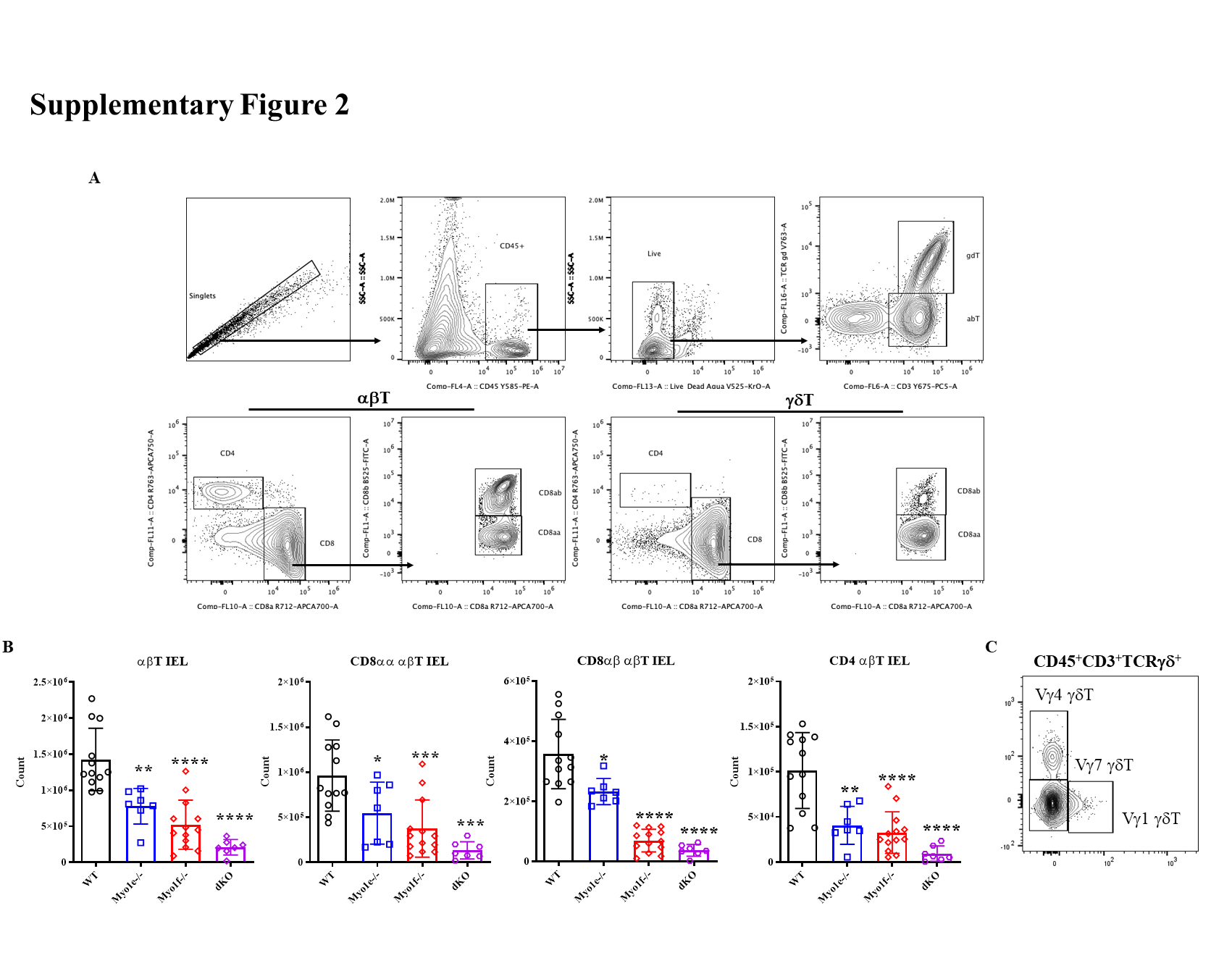


**Figure supplementary 2. Gate strategy analysis of αβT and γδT IEL analysis and αβT IEL count. A)** Gate strategy analysis employed to count αβT and γδT IEL subpopulations. **B)** Total αβT, CD8αα αβT, CD8αβ αβT and CD4 αβT IEL count in WT, Myo1e-/-, Myo1f-/- and dKO mice. **C)** Representative dot plot of Vγ-specific γδT IEL analysis. Each dot represents one mouse. *t*-student was applied, p value; *=0.05, **=0.005, ***=0.0005, ****=0.0001.


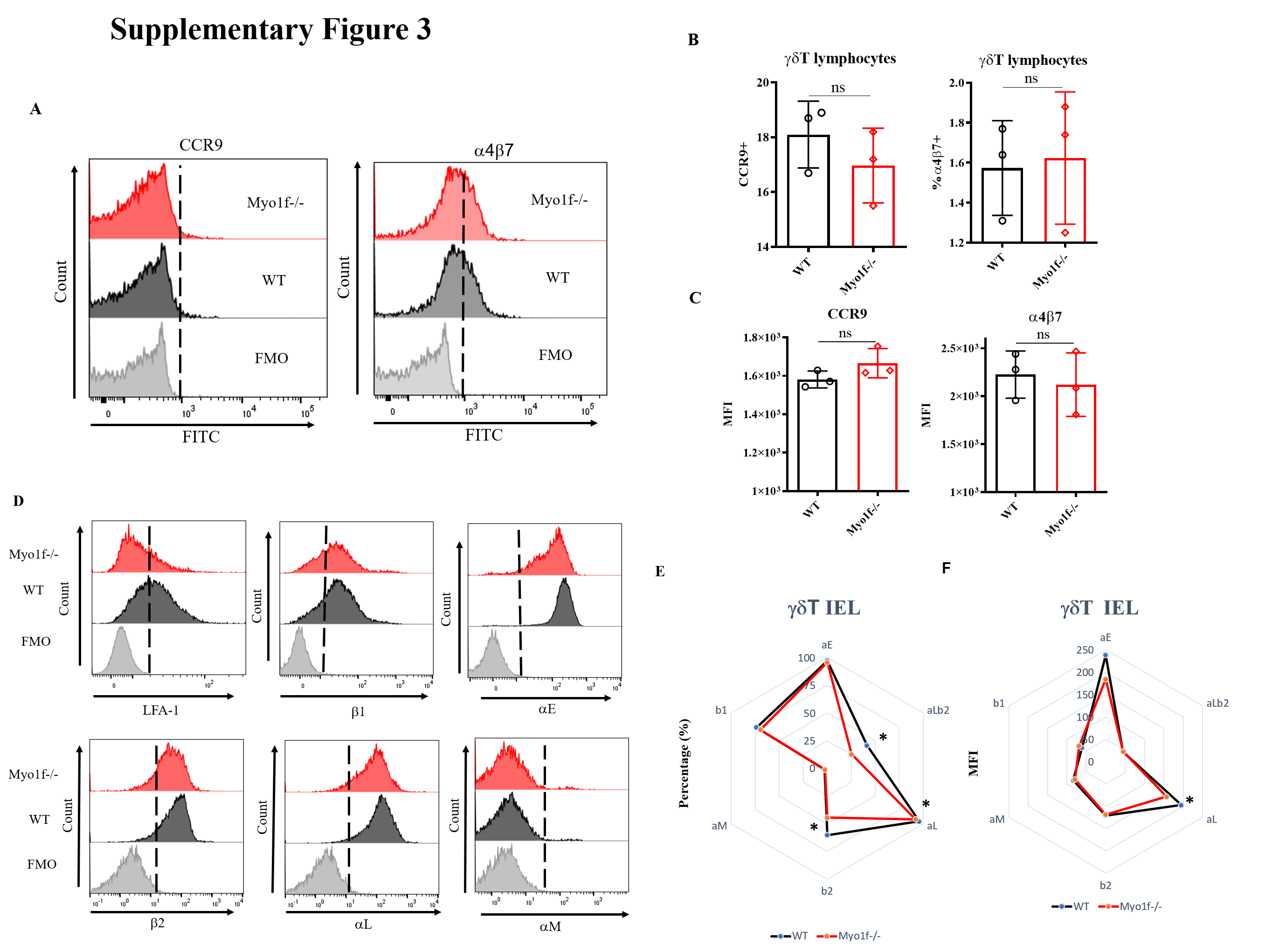


**Supplementary figure 3. Gut homing receptors expression in thymus γδT lymphocytes. A)** Representative histograms of CCR9 and α4β7 expression in thymus γδT lymphocytes from WT and Myo1f-/- mice. **B)** Percentage of CCR9 and α4β7 positive thymus γδT lymphocytes. **C)** MFI of surface CCR9 and α4β7 expression. **D)** Representative histograms of αLβ2, αL, β2, αM, αE, and β1 integrin expression. **E)** Radar plot of the proportion of γδT cell integrin positive cells. **F)** Radar plot of MFI of integrin+ γδ T cells. Three independent experiments. In radar plots, each plot represents the mean value. WT vs. Myo1f-/- comparison was applied by the t-Student test.


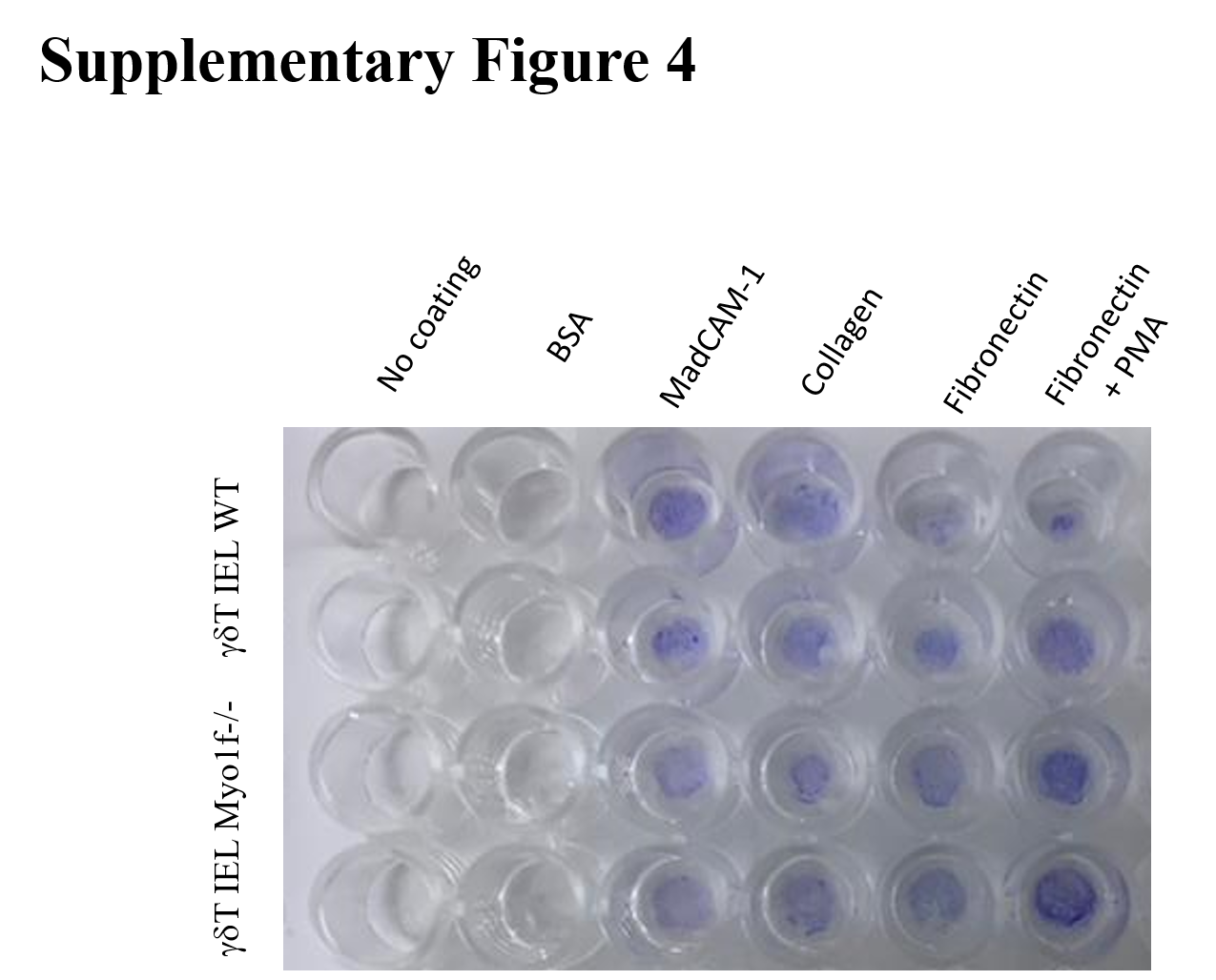


**Supplementary figure 4. Crystal violet staining of cell adhesion assay.**


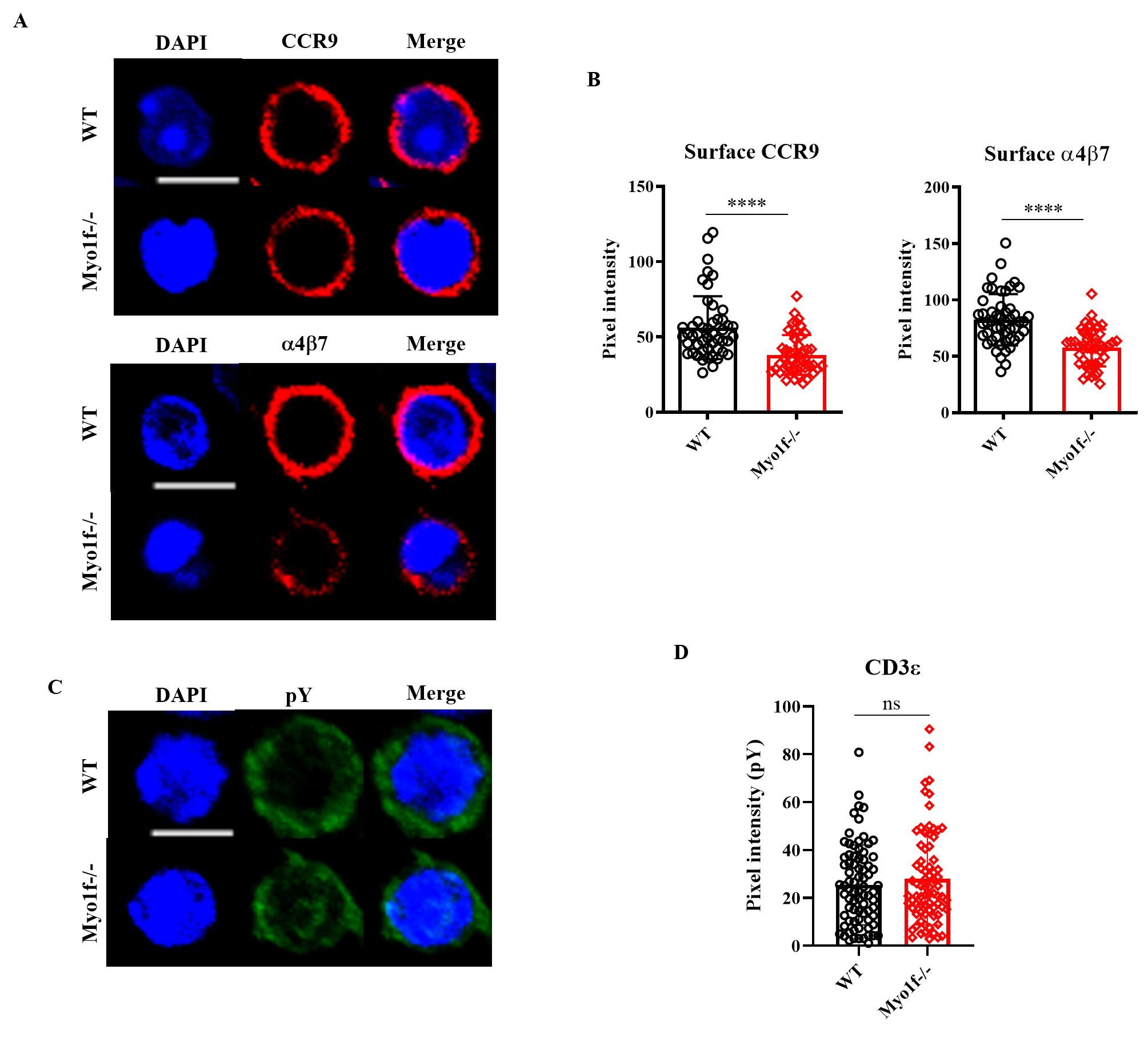


**Supplementary figure 5. CCR9 and α4β7 surface localization and CD3ε induced phosphorylation. A)** Representative cells showing CCR9 and α4β7 localization in γδT IEL from WT and Myo1f-/- mice. **B)** Pixels intensity of CCR9 and α4β7 positive γδT IEL. **C)** Representative cells showed tyrosine phosphorylation induced by CD3ε. **D)** Pixels intensity of tyrosine phosphorylation by CD3ε stimulation. *t*-student was applied.

**Supplementary movie 1. Random migration of WT γδT IEL.** 2 x 10^5^ WT γδT IEL were seeded in a collagen IV coated μ-Slide chemotaxis chamber for 30 min at 37 ºC. Non-adhered cells were removed and the chamber was filled with complete RPMI-1640 medium without any recombinant chemokine. Time-lapse videos were taken every 30 sec for 30 min and analyzed with Fiji software. Movie is presented as AVI (Audio Video Interleave) at 10 frames per second.

**Supplementary movie 2.** **Random migration of Myo1f-/- γδT IEL.** 2 x 10^5^ Myo1f-/- γδT IEL were seeded in a collagen IV coated μ-Slide chemotaxis chamber for 30 min at 37 ºC. Non-adhered cells were removed and the chamber was filled with complete RPMI-1640 medium without recombinant chemokine. Time-lapse videos were taken every 30 sec for 30 min and analyzed with Fiji software. Movie is presented as AVI (Audio Video Interleave) at 10 frames per second.

**Supplementary movie 3. CCL25-dependent migration of WT γδT IEL.** 2 x 10^5^ WT γδT IEL were seeded in a collagen IV coated μ-Slide chemotaxis chamber for 30 min at 37 ºC. Non-adhered cells were removed and the chamber was filled with complete RPMI-1640 medium. Recombinant CCL25 chemokine (100 ng/mL) was added in the left side plug immediately before the imaging. Time-lapse videos were taken every 30 sec for 30 min and analyzed with Fiji software. Movie is presented as AVI (Audio Video Interleave) at 10 frames per second.

**Supplementary movie 4. CCL25-dependent migration of Myo1f-/- γδT IEL.** 2 x 10^5^ Myo1f-/- γδT IEL were seeded in a collagen IV coated μ-Slide chemotaxis chamber for 30 min at 37 ºC. Non-adhered cells were removed and the chamber was filled with complete RPMI-1640 medium. Recombinant CCL25 chemokine (100 ng/mL) was added in the left side plug immediately before the imaging. Time-lapse videos were taken every 30 sec for 30 min and analyzed with Fiji software. Movie is presented as AVI (Audio Video Interleave) at 10 frames per second.

**Supplementary movie 5.** **CCL25-dependent migration of WT + α-CCR9 antibody γδT IEL.** 2 x 10^5^ WT γδT IEL previously incubated with anti-CCR9 antibody (5 μg/ml, clone 9B1) for 1 h at 37 ºC, were seeded in a collagen IV coated μ-Slide chemotaxis chamber for 30 min at 37 ºC. Non-adhered cells were removed and the chamber was filled with complete RPMI-1640 medium. Recombinant CCL25 chemokine (100 ng/mL) was added in the left side plug immediately before the imaging. Time-lapse videos were taken every 30 sec for 30 min and analyzed with Fiji software. Movie is presented as AVI (Audio Video Interleave) at 10 frames per second.
